# Commentary: Playing the best hand in conduit selection

**DOI:** 10.1016/j.xjtc.2021.10.012

**Published:** 2021-10-19

**Authors:** David L. Joyce

**Affiliations:** Department of Surgery, Medical College of Wisconsin, Milwaukee, Wis

**Figure:**
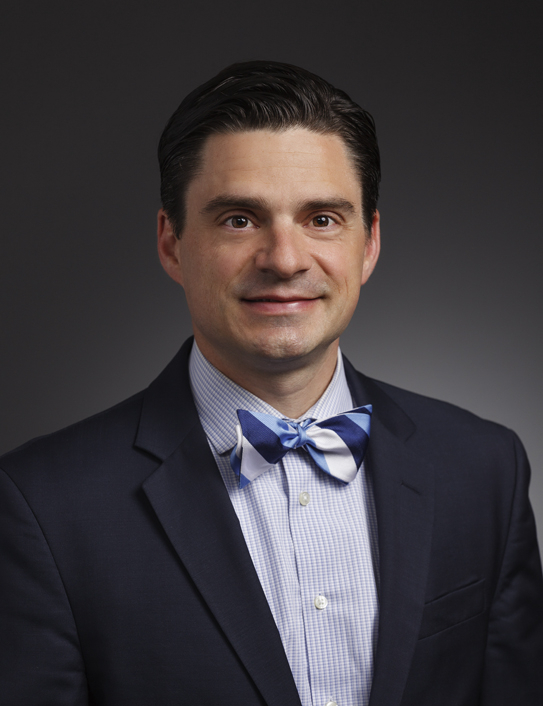
David L. Joyce, MD, MBA, FACS

**Central Message**

The growing evidence in support of using the radial artery as a preferred “second” conduit should be carefully considered by all CABG surgeons.

See Article page 114.

The short answer to the question “When and when not?” with respect to using a radial artery for coronary artery bypass grafting (CABG) is “it depends.” The Weill Cornell group has beautifully outlined each of the important factors to consider when answering this question, and every coronary surgeon should become well acquainted with this analysis.^1^ The authors provide an evidence-based rationale for considering the radial artery as the default option in “second” conduit selection. The Radial Artery Patency and Clinical Outcomes (RAPCO) data in particular demonstrate both survival and 10-year patency advantages of choosing a radial artery over the right internal thoracic artery.^2^

While the manuscript is silent on the question of current use rates, they are likely to be in the single digits. The authors describe a comprehensive list of contraindications, but these likely only provide part of the explanation. Whether through open or endoscopic harvesting techniques, the skill of the operator must surely be considered when taking this approach. For surgeons who are not blessed with an experienced radial harvester in their practice, the adoption of this technique comes with considerable switching costs. Choreography also comes into play when the disease complexity requires more than 2 grafts. When adding a third or fourth graft to the equation (which is the rule rather than the exception), there is an increasing temptation to consider using the saphenous vein. By the time the endoscope has found its way into the patient's thigh, the cost/benefit analysis of acquiring the third conduit has changed, and interpretation of the Radial-Artery or Saphenous-Vein Grafts in Coronary-Artery Bypass Surgery (RADIAL) trial begins to focus on the similar incidence of death between groups.^3^

For those not persuaded by the scientific data on this topic, consider the matter purely from a self-serving perspective that seeks to optimize only the benefits derived by the surgeon. Suppose that in your practice you have 4 assistants who can harvest vein. One of them is also trained in radial artery preparation techniques. A reasonable assumption would be that the assistant who has gone to the trouble to learn radial harvesting might have slightly superior skills to those that were not afforded this opportunity. Another reasonable assumption might be that skills in conduit acquisition serve as a marker for other important skills in a CABG operation, such as target vessel exposure or overall dexterity. If you announce tomorrow that the recent *JTCVS Techniques* paper has persuaded you to use a radial artery on all CABG procedures, you've just deftly secured the best assistant in your practice. If the patient doesn't have any suitable vein, you get an extra conduit out of the deal. At some point a Nash equilibrium will occur where all the surgeons demand a radial, but that could take years to unfold. My own decision to switch to all radials is obviously based purely on scientific data, but I also won't deny the value of having a highly skilled assistant in every case.
